# The effect of industry-related air pollution on lung function and respiratory symptoms in school children

**DOI:** 10.1186/s12940-018-0373-2

**Published:** 2018-03-27

**Authors:** Arnold D. Bergstra, Bert Brunekreef, Alex Burdorf

**Affiliations:** 1 Department of Public Health, Erasmus MC, University Medical Centre, PO Box 2040, 3000CA, Rotterdam, the Netherlands; 2 Institute for Risk Assessment Sciences, Utrecht University, PO Box 80176, 3508TD, Utrecht, the Netherlands; 3 The Zeeland Public Health Service, PO Box 345, 4460AS, Goes, the Netherlands; 4 Julius Center for Health Sciences and Primary Care, University Medical Center Utrecht, P.O. Box 85500, 3508 GA Utrecht, The Netherlands

**Keywords:** Air pollution, Heavy industry, Respiratory symptoms, Lung function, School children

## Abstract

**Background:**

Heavy industry emits many potentially hazardous pollutants into the air which can affect health. However, the effects of air pollution from heavy industry on lung function and respiratory symptoms have been investigated scarcely. Our aim was to investigate the associations of long-term air pollution from heavy industry with lung function and respiratory symptoms in school children.

**Methods:**

A cross-sectional lung function study was conducted among school children (7–13 years) in the vicinity of an area with heavy industry. Lung function measurements were conducted during school hours. Parents of the children were asked to complete a questionnaire about the health of their children. A dispersion model was used to characterize the additional individual-level exposures to air pollutants from the industry in the area. Associations between PM_2.5_ and NO_X_ exposure with lung function and presence of respiratory symptoms were investigated by linear and/or logistic regression analysis.

**Results:**

Participation in the lung function measurements and questionnaires was 84% (665/787) and 77% (603/787), respectively. The range of the elevated PM_2.5_ and NO_X_ five years average concentrations (2008–2012) due to heavy industry were 0.04–1.59 μg/m^3^ and 0.74–11.33 μg/m^3^ respectively. After adjustment for confounders higher exposure to PM_2.5_ and NO_X_ (per interquartile range of 0.56 and 7.43 μg/m^3^ respectively) was associated with lower percent predicted peak expiratory flow (PEF) (B -2.80%, 95%CI -5.05% to − 0.55% and B -3.67%, 95%CI -6.93% to − 0.42% respectively). Higher exposure to NO_X_ (per interquartile range of 7.43 μg/m^3^) was also associated with lower percent forced vital capacity (FVC) and percent predicted forced expiration volume in 1 s (FEV1) (B -2.30, 95% CI -4.55 to − 0.05 and B -2.73, 95%CI -5.21 to − 0.25 respectively). No significant associations were found between the additional exposure to PM_2.5_ or NO_X_ and respiratory symptoms except for PM_2.5_ and dry cough (OR 1.40, 95%CI 1.00 to 1.94).

**Conclusion:**

Exposure to PM_2.5_ and NO_X_ from industry was associated with decreased lung function. Exposure to PM_2.5_ was also associated with parents’ reports of dry cough among their children.

**Electronic supplementary material:**

The online version of this article (10.1186/s12940-018-0373-2) contains supplementary material, which is available to authorized users.

## Background

Air pollution is a complex mixture of different gaseous and particulate components and can cause several health effects. Both long- and short-term exposure to air pollution can cause cardiovascular diseases, respiratory diseases (e.g. asthma, chronic obstructive pulmonary disease) and mortality [1]. Children are more susceptible to the effects of air pollution than adults. The lack of a fully developed pulmonary metabolic capacity in children make them more susceptible to air pollutants compared with adults [2]. Moreover, children are in general more exposed because of greater physical activity of children compared with adults, as well a greater time spent out of doors.

Investigations often focus on emissions from road traffic, smog and urban or regional differences in air pollution. The influence of air pollution from heavy industry on lung function or respiratory symptoms is less often explored [3–6]. The impact of localised air pollution from industry on health is a major concern in some areas. However, it is often a problem to disentangle the effects of the exposure of traffic from exposure of industry.

Lung function is unlike respiratory symptoms an objective measure of respiratory health. Some studies have observed a reduction in lung function or a higher prevalence of respiratory symptoms among children living in the neighbourhood of industry compared to a control area while other studies found no association. In Canada a cross-sectional study among children (aged 6 to 18 years) found a significant reduction of 1% in predicted FEV1 (1-s forced expiratory volume) due to an increase of 190 t of industrial air PM_2.5_ (particulate matter with an aerodynamic diameter < 2.5 μm) emissions within 25 km of residence. This association was only observed among boys, but not among girls [7]. A cross-sectional study in Argentina children (aged 6 to 12 years) living near petrochemical industry had a lower lung function (13% FEV1 percent predicted) and significant more asthma (24.8% vs 10.1%), asthma exacerbations (6.7 vs 2.9 per year) and respiratory symptoms (average 24.4% vs 14.0%) compared to children in a semirural region [8]. In Italy a cross-sectional study among children (aged 6 to 14 years) living in the vicinity of petrochemical industry showed a lower lung function (10.3% FEV1 and 12.9% MMEF (maximum midexpiratory flow)) and an increase in wheezing symptoms (adjusted prevalence ratio of 1.70) compared to children in a reference area [9]. A Spanish cross-sectional study among children (aged 13 to 14 years) living in the neighbourhood of petrochemical industry versus children with no industry in surrounding areas found no significant associations between exposure and lung function or respiratory symptoms [10].

To the best of our knowledge, studies about the association between industry-related air pollution and health among children are rare. Therefore, the aim of this study was to investigate the effect of air pollution from industry on lung function and respiratory symptoms in children.

## Methods

### Study design and population

A cross-sectional study was conducted among school children (aged 7–13 years) in the vicinity (about 2–35 km) of the large industrial areal (Sloe area) near East Vlissingen in the Southwest of the Netherlands. At the time of the study several heavy industries were active in the area such as a coal power plant, terminals for storing and shipping of coal, a plastic recycling company, a phosphorus chemical company, an oil refinery and an aluminium smelter.

The parents of the school children received an invitation letter with a consent form for conducting a lung function of their child and also a request to complete an online questionnaire on their child’s health by using a login code provided in the letter. The invitation letters were distributed by the school of the child. Two reminders were sent in case of non-response.

The lung function measurements were conducted at school from 19 November to 9 December 2012. The questionnaires were collected from 15 November 2012 to 1 February 2013.

### Exposure assessment

A variety of components were emitted by plants in the industrial area near East Vlissingen in the Southwest of the Netherlands like particulate matter (PM), nitrogen oxide (NO_X_), sulphur dioxide (SO_2_), ethylene, formaldehyde, toluene, benzene, and dioxins. It is difficult to define an exposure measure of relevance when the biological mechanisms are largely unknown. Moreover, the air pollution mix varied greatly by locality and time [11]. For this study relevant compounds were selected in two steps.

First, the emission (kg/year) of a compound was divided by the European Commission limit values or if not available the maximum permissible risk levels (MPR) in air (μg/m^3^) from the National Institute for Public Health and the Environment, The Netherlands (RIVM). The compounds with a high fraction (more than 5000) were selected. Next, the annual mean concentrations of these compounds were estimated with a dispersion model. The compounds with the highest scatter were selected.

The emission data was obtained from the Emission Register [12]. The Netherlands National Institute for Public Health and the Environment (RIVM) co-ordinates the annual compilation of the Emission Register on behalf of the Dutch Ministry of Infrastructure and Environment. Emission factors are derived from measurements and calculations of a model or from (the international) literature.

The Operational Priority Substances (OPS) dispersion model (version 4.5.0) [13], developed by the Netherlands National Institute for Public Health and the Environment (RIVM), was used to calculate concentration levels at individual homes. The OPS model requires emission data (emission strength, emission height, coordinates source, heat capacity and substance) and hourly-based meteorological data (among others: temperature, relative humidity, wind speed, wind direction, precipitation and global/solar) as input for the calculations. The meteorological data were retrieved from the Royal Netherlands Meteorological Institute (KNMI). The OPS model also requires a receptor file. The geographic information system QGIS (version 2.18.0) was used to geocode (by means of a plugin) the home and school addresses of the children. The x,y coordinates of the home and schools addresses were used for the receptor file in the OPS model. Thus, the dispersion model estimates the exposure to specific compounds attributable to industry, in addition to background exposure due to other exposure sources including traffic and agriculture. After the dispersion calculations the air pollution data were linked to the lung function data and questionnaires by means of a Trusted Third Party to ensure confidentiality of personal information.

Dutch law requires primary schoolchildren to attend classes for 940 h a year. A time weighted average exposure was calculated taking in account the time and exposure at school (940 h a year) and at home (7820 h a year). Holiday time, travel time to school and time spent on sports were not included in the time weighted average exposure calculation.

After applying the first selection step (mentioned before) the following compounds remained: PM_2.5_ (particulate matter with an aerodynamic diameter < 2.5 μm), PM_10_ (particulate matter with an aerodynamic diameter < 10 μm), SO2, and NO_X_. These compounds were highly correlated (Pearson correlation coefficients ranged from 0.88 to 0.996). Because of the high correlation, associations between the outcomes and these four components cannot be disentangled. NO_X_ was chosen because it had the largest scatter of the four compounds. PM_10_ and PM_2.5_ have the lowest correlation with NO_X_. PM_10_ en PM_2.5_ also have a similar scatter. Therefore, the average concentration of PM_10_ and PM_2.5_ was compared with the European Commission limit value by dividing the average concentration by the limit value. PM_2.5_ was chosen because it has a higher fraction than PM_10_.

Because of the high correlation between the time weighted average exposure of PM_2.5_ and NO_X_ (Pearson correlation of 0.88), the components must be regarded as indicators of the mixture of air pollution rather than particular causative factors of adverse health effects.

In the study area the exposure to air pollution form traffic is relatively low (less than 5000 vehicles per day or the distance between road and house is more than 100 m). Only three (trunk) roads (A58, N62 and N254) have more than 5000 vehicles per day (35,000, 18,000 and 12,000 vehicles per day respectively). To avoid interference of traffic exposure, cases were excluded from the analysis if the distance between these three roads and a child’s home address was less than 100 m.

### Lung function

School children aged 7–13 years underwent an examination of the lung function by one of the two experienced operators, each using one of the two portable spirometers (EasyOne, NDD Medical Technologies, Zürich, Switzerland). Quality control assessment was done electronically (software spirometer) and manually. End-of-Test criteria, quality criteria and quality grading in EasyOne-PC were based upon published standards [14–17]. Lung function measurement that met the quality criterion of at least 2 acceptable tests and a difference between the best two FEV1 and FVC values equal to or less than 200 ml were selected.

The lung function tests were reviewed by a pulmonary function technician who made the final decision on acceptance or rejection. The following variables were obtained from the current analysis: forced vital capacity (FVC), 1-s forced expiratory volume (FEV1), peak expiratory flow (PEF), the maximum midexpiratory flow (MMEF) also known as forced expiratory flow between the 25th and 75th percent of FVC (FEF25–75) and the FEV1/FVC ratio, also called Tiffeneau-Pinelli index. To calculate the predicted lung function, the weight and height of the children were measured. Internal prediction formulas were developed. The natural logarithms of lung function variables were regressed on the logarithms of age and weight, and an interaction between sex and the logarithm of height [18]. In addition also low lung function, defined as < 85% of the internal predicted value, was calculated.

Lung function measurements were conducted only on days when the school had not been downwind from the industry for at least two days, to avoid acute effects of air pollutants on the days of the examinations.

### Questionnaire on health and risk factors

The questionnaire consisted of four parts namely: socio-demographic characteristics, (respiratory) health problems of the child, indoor air pollution and family history for asthma predisposition.

#### Socio-demographics

Demographic characteristics were gender and age of the school children (categorized in: 7–8, 9–10 and 11–13 years). The question about education level of the parents was categorized in: 1) primary school or less (8 years of education or less), 2) lower general secondary education (12 years of education), 3) higher general secondary education (14 years of education) and 4) college or university (more than 14 years of education). The highest educational level of the parents was used as indicator.

#### Respiratory symptoms

For the questionnaire about the respiratory symptoms of the children the core questions from the International Study on Asthma and Allergies in Children (ISAAC) were used [19–21]. These questions were 1) Has your child had wheezing or whistling in the chest in the past 12 months? 2) In the past 12 months, has your child’s chest sounded wheezy during or after exercise? 3) In the past 12 months, has your child had a dry cough at night, apart from a cough associated with a cold or a chest infection? Reported “asthma” was defined from the question “Has your child ever had asthma?”. We defined a current asthma case as a child who ever had asthma and wheeze in the past 12 months. In addition to respiratory symptoms also questions about allergy to dust mite and animals has been asked (yes/no).

#### Proportion time exposed

The time a child was exposed was assessed by how many years the family have lived at the current address in the past five years.

#### Indoor pollution

Passive smoking was assessed by whether a family member smoked in house. After the following sources of indoor air have been asked: use of a wood stove, having domestic pets and molds in the living and/or sleeping room (yes/no). Sufficient ventilation was measured with the question: Can you indicate how long the living room is ventilated during winter? If the room was not continuous ventilated, the ventilation was categorized as insufficient.

#### Family history for asthma predisposition

The family history of asthma was measured with the question: Has one or more people in the family ever suffered from asthma?

### Statistical analyses

Multivariate linear and logistic regression analyses were performed to control for potential confounders. There were two models for each lung function parameter and respiratory symptom. Model 1 was the adjusted model for gender and age; Model 2 added education parents, molds, passive smoking, allergy, ventilation, fireplace, pets, proportion time exposed and family history for asthma predisposition. In the statistical models with a lung function parameter as dependent variable, adjustment was also done for possible differences between the two operators. When a potential confounder had a *p*-value greater than 0.3, then this variable was removed from the final model. The variables gender, age and operator (if applicable) were included in the model by default.

Because of non-responders to the questionnaire, the analyses with model 2 were conducted with a smaller study population than the analyses with model 1. Sensitivity analyses were conducted to evaluate whether the reduction of the study population had any influence on the reported associations.

The statistical analyses were conducted with the statistical package IBM SPSS version 21 (SPSS Inc., Chicago, IL, USA). Results are presented with 95% confidence intervals (CI). A *p* value less than 0.05 was considered to be statistically significant.

## Results

In total, 665 of the 787 school children aged 7–13 years underwent an examination of the lung function (response 84%). Parents filled in 603 questionnaires after two reminders (which results in an overall response of 77%). Children living near a busy road were excluded (11 cases). Of the remaining children 559 had a lung function measurement that satisfied the quality criteria (see methods). Among parents 594 persons had a completed questionnaire. Hence, the study population consisted of 424 children-parent combinations with complete information on both lung function and respiratory symptoms.

**Fig. 1:**
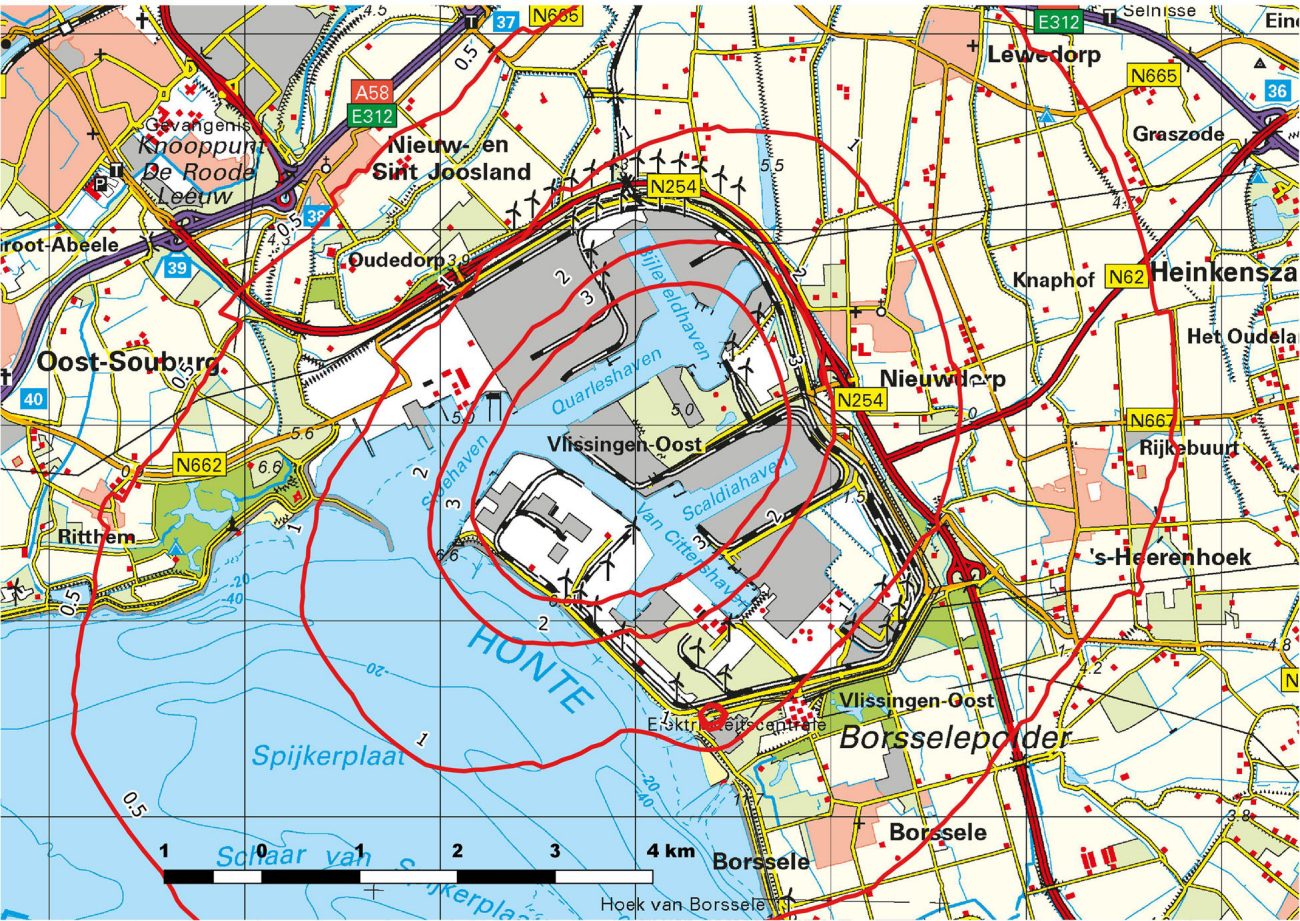
Modelled PM_2.5_ isoconcentration contours (μg/m^3^), five years average exposure (2008–2012) without background concentration (map reprinted from Kadaster [28] in the Netherlands under a CC-BY-4.0 license, 2017)

Table 1 shows the characteristics of the children, parents and their exposure. The exposure patterns showed that most persons in the study population had a modestly increased additional exposure to PM_2.5_ and NO_X_ from industrial emissions. This is also reflected in the five years (2008–2012) average iso-concentration contours of PM_2.5_ and NO_X_ without background concentration (see Fig. 1 and Additional file 1: Figure S1 respectively).

**Table 1:** Characteristics of school children and their environment (*n* = 594)

Characteristic (SD)	
Gender (male %)	51
Age groups (%)	
7–8 years	28
9–10 years	36
11–13 years	36
Average height (cm)	141 (11)
Allergies (%)	8
Parental education (%)	
Primary school	0.2
Lower general secondary education	18
Higher general secondary education	49
College, university	33
Passive smoking in house	11
Others members of the family has asthma	21
Indoor air pollution (%)	
Ventilation insufficient	67
Wood burning stove	28
Damp or mold	12
Pets	58
Exposure to outdoor PM_2.5_ concentration (2008–2012)^a^ (μg/m^3^)	
Median	0.37
Interquartile range	0.56
Minimum – Maximum	0.04–1.59
Exposure to outdoor NO_X_ concentration (2008–2012)^a^ (μg/m^3^)	
Median	2.50
Interquartile range	7.43
Minimum – Maximum	0.74–11.33
Time exposed to outdoor pollution (%)	
Five years or more	88

^a^Without background concentration

Table 2 shows that the lung function parameters were comparable to the expected values from the reference equations. In this table also z-score, using the Global Lung Function (GLI) reference values [22], were added to make comparison possible with other study populations. The PEF and MMEF showed the highest prevalence of low lung function. The most common respiratory symptom among children was dry cough followed by wheezing, wheezing during exercise, and asthma.

**Table 2:** Prevalence of respiratory symptoms in school children (7–13 years) and the lung function among these children

Prevalence of respiratory symptoms (*n* = 594)	
Wheezing (%)	7
Wheezing during exercise (%)	5
Asthma (%)	4
Dry cough (%)	20
Average percent predicted spirometric lung function (*n* = 559)	
FVC (% predicted, SD)	101 (11)
FEV1 (% predicted, SD)	101 (12)
PEF (% predicted, SD)	101 (16)
MMEF (% predicted, SD)	103 (25)
FEV1/FVC (% predicted, SD)	100 (7)
Low FVC (%)^a^	7
Low FEV1 (%)^a^	9
Low PEF (%)^a^	15
Low MMEF (%)^a^	22
Low FEV1/FVC (%)^a^	3
Z-score FVC^b^	0.173
Z-score FEV1^b^	−0.070
Z-score MMEF^b^	−0.404
Z-score FEV1/FVC^b^	−0.448

^a^< 85% predicted ^b^GLI reference

Tables 3 and 4 shows that children exposed to PM_2.5_ and NO_X_ (per interquartile range of 0.56 and 7.43 μg/m^3^ respectively) had a significantly lower percent predicted peak expiratory flow (PEF) (B -2.80%, 95%CI -5.05% to − 0.55% and B -3.67%, 95%CI -6.93% to − 0.42% respectively). Children exposed to NO_X_ (per interquartile range of 7.43 μg/m^3^) also had a significantly lower percent forced vital capacity (FVC) and percent predicted 1-s forced expiratory volume (FEV1) (B -2.30, 95%CI -4.55 to − 0.05 and − 2.73 95%CI -5.21 to − 0.25 respectively). Gender and age were not significant associated with the percent predicted FVC, FEV1 and PEF. After adjustment for gender, age and operator, exposure to PM_2.5_ and NO_X_ (per interquartile range of 0.56 and 7.43 μg/m^3^ respectively) was significantly associated with a low PEF (OR 1.42, 95%CI 1.01 to 1.99 and OR 1.75, 95%CI 1.07 to 2.87 respectively). With further adjustment for confounders no significant association was found.

**Table 3:** Associations between long-term exposure (2008–2012) to PM_2.5_ and NO_X_ (per interquartile range) and predicted lung function among school children (7 to 13 years) in linear regression analysis

	FVC (% predicted)	FEV1 (% predicted)	PEF (% predicted)	MMEF (% predicted)	FEV1/FVC (% predicted)
	B (95% CI)	B (95% CI)	B (95% CI)	B (95% CI)	B (95% CI)
Model 1^a^ (n = 559)					
PM_2.5_ (0.56 μg/m^3^)	−0.14 (− 1.53–1.25)	−0.34 (− 1.85–1.18)	−3.04 (− 5.05 - -1.02)**	−1.10 (− 4.21–2.00)	−0.24 (− 1.10–0.63)
NO_X_ (7.43 μg/m^3^)	− 1.07 (− 3.04–0.90)	−1.41 (− 3.56–0.74)	−4.71 (− 7.56 - -1.86)**	−2.22 (− 6.62–2.19)	−0.35 (− 1.58–0.89)
Model 2^b^ (*n* = 424)					
PM_2.5_ (0.56 μg/m^3^)	−0.76 (−2.32–0.79)	−1.15 (− 2.86–0.56)	−2.80 (− 5.05 - -0.55)*	−1.29 (− 4.68–2.10)	−0.29 (− 1.26–0.69)
NO_X_ (7.43 μg/m^3^)	− 2.30 (− 4.55 - -0.05)*	−2.73 (− 5.21 - -0.25)*	− 3.67 (− 6.93 - -0.42)*	−2.80 (− 7.69–2.09)	−0.35 (− 1.75–1.06)

^a^ Adjusted for gender, age and operator

^b^Adjusted for gender, age, education parents, molds, passive smoking, allergy, ventilation, fireplace, pets, family history for asthma predisposition, proportion time exposed in the last five years and operator (smaller study population due to non-responders of the questionnaire)

*P < 0.05, ** *p* < 0.01, *** *p* < 0.001

**Table 4:** Associations between long-term exposure (2008–2012) to PM_2.5_ and NO_X_ (per interquartile range) and low lung function among school children (7 to 13 years) in logistic regression analysis

	Low FVC^c^	Low FEV1^c^	Low PEF^c^	Low MMEF^c^	Low FEV1/FVC^c^
	OR (95% CI)	OR (95% CI)	OR (95% CI)	OR (95% CI)	OR (95% CI)
Model 1^a^ (n = 559)					
PM_2.5_ (0.56 μg/m^3^)	1.05 (0.64–1.70)	1.07 (0.69–1.65)	1.42 (1.01–1.99)*	1.00 (0.74–1.36)	0.97 (0.43–2.20)
NO_X_ (7.43 μg/m^3^)	0.99 (0.49–1.99)	1.04 (0.56–1.95)	1.75 (1.07–2.87)*	1.13 (0.73–1.73)	1.17 (0.40–3.44)
Model 2^b^ (n = 424)					
PM_2.5_ (0.56 μg/m^3^)	1.34 (0.80–2.24)	1.26 (0.79–2.01)	1.37 (0.95–1.97)	1.00 (0.71–1.40)	0.88 (0.34–2.25)
NO_X_ (7.43 μg/m^3^)	1.43 (0.66–3.12)	1.41 (0.70–2.86)	1.63 (0.94–2.82)	1.10 (0.68–1.79)	0.95 (0.27–3.40)

^a^Adjusted for gender, age and operator

^b^Adjusted for gender, age, operator, education parents, molds, passive smoking, allergy, ventilation, fireplace, pets, family history for asthma predisposition, proportion time exposed in the last five years and operator (smaller study population due to non-responders of the questionnaire)

^c^< 85% predicted

*P < 0.05, ** *p* < 0.01, *** *p* < 0.001

In Table 5 it is shown that odds ratios for the relationship between industry-related exposure (PM_2.5_ and NO_X_) and respiratory symptoms among children, with the exception of asthma, were all elevated. Only exposure to PM_2.5_ was statistically significant elevated with the respiratory symptom ‘dry cough’ (OR 1.40, 95%CI 1.00 to 1.94).

**Table 5:** Associations between long-term exposure (2008–2012) to PM_2.5_ and NO_X_ (per interquartile range) and respiratory symptoms among school children (7 to 13 years) in logistic regression analysis

	Wheezing OR (95% CI)	Wheezing during exercise OR (95% CI)	Asthma OR (95% CI)	Dry cough OR (95% CI)
Model 1^a^ (*n* = 594)				
PM_2.5_ (0.56 μg/m3)	1.33 (0.84–2.11)	1.29 (0.74–2.25)	0.97 (0.49–1.92)	1.29 (0.95–1.74)
NO_X_ (7.43 μg/m3)	1.25 (0.64–2.46)	1.20 (0.53–2.74)	0.73 (0.28–1.95)	1.49 (0.97–2.28)
Model 2^b^ (*n* = 506)				
PM_2.5_ (0.56 μg/m^3^)	1.58 (0.93–2.68)	1.43 (0.78–2.61)	1.05 (0.50–2.19)	1.40 (1.00–1.94)*
NO_X_ (7.43 μg/m^3^)	1.35 (0.61–3.00)	1.25 (0.51–3.04)	0.76 (0.25–2.32)	1.50 (0.92–2.43)

^a^Adjusted for gender and age

^b^Adjusted for gender, age, education parents, molds, passive smoking, allergy, ventilation, fireplace, pets, family history for asthma predisposition, proportion time exposed in the five years and asthma predisposition (smaller study population due to non-responders of the questionnaire)

*P < 0.05, ** *p* < 0.01, *** *p* < 0.001

## Discussion

This study showed that higher exposure to PM_2.5_ and NO_X_ from industrial sources (per interquartile range of 0.56 and 7.43 μg/m^3^ respectively) was significantly associated with lower percent predicted PEF of 2.80% and 3.67% respectively. Higher NO_X_ exposure (per interquartile range of 7.43 μg/m^3^) was also significantly associated with 2.30% and 2.73% lower percent predicted FVC and FEV1 respectively.

The odds ratios for the relationship between industry-related exposure (PM_2.5_ and NO_X_) and respiratory symptoms among children, with the exception of asthma, were all elevated. Only higher exposure to PM_2.5_ was significant associated with a 1.40 higher odds ratio of dry cough.

In 2011 a cross-sectional questionnaire study among children between 2 and 18 years was conducted in the same area which showed that higher PM_2.5_ and NO_X_ concentrations, as predicted by an exposure dispersion model, were statistical significant associated with an excess of wheezing and dry cough [23]. In the current study we observed similar associations between PM_2.5_ and NO_X_ (per μg/m^3^) exposure and the presence of respiratory symptoms with a statistically significant association between PM_2.5_ and dry cough. In the previous study in 2011 PM_2.5_ and NO_X_ were significantly associated with presence of wheezing, wheezing during exercise, and dry cough. Due to the smaller study population in the current study compared to the study in 2011 (594 vs 1099) associations of similar magnitude lacked sufficient power.

Comparable studies about the influence of industry-related air pollution on lung function among children are rare. To the best of our knowledge there are no other studies which describe association of modelled PM_2.5_ or NO_X_ exposure from industry and lung function level. Other studies have compared populations living in industrial areas with control areas or have relied on emission information instead of modelled exposure patterns. Several studies on the effects of air pollution from traffic on lung function have reported lower lung function with higher exposure to air pollution [24].

The population’s mean lung function decrement from exposure to PM_2.5_ and NO_X_ is relative small, but this was not the case for the percentage of children with a poor lung function. We found that a 3% decrease in predicted PEF from exposure to PM_2.5_ (in model 1, adjusted for gender, age and operator) corresponds to a 40% higher odds ratio for a low PEF (< 85% predicted). A 5% decrease in predicted PEF (in model 1) from exposure to NO_X_ corresponds to a 75% higher odds ratio for a low PEF. Thus, small decreases in the mean in the general population of healthy school children are associated with a relevant increase in the number of children with a poor lung function.

This study has certain strengths and limitations. First, a strength of the study is the use of lung function as objective measure of respiratory health. A second strength is that exposure to air pollution was based on a dispersion model. Good agreement was found for both SO_X_ and NO_X_ between modelled and measured concentrations for the OPS dispersion model [13]. A dispersion model takes factors, such as stack height, exact distance between stack and the home of the children, weather and climate, into account. A limitation is the use of two components as indicators for the exposure to air pollution. A variety of components were emitted by the industry in the industrial area. The exposure of each component may vary by locality. Moreover, the different components can have an additive, synergistic or antagonistic effect. Because of the high correlation between PM_2.5_ and NO_X_, it was not possible to single out association specific to a particular air pollution component.

A second limitation is that most of the children were exposed for five or more years (88%). Also the proportion time exposed in the past five years was not associated with the percent predicted FVC, FEV1 and PEF. In this study the effect of the moment (e.g. current year) of the exposure and duration could therefore not be disentangled.

A third limitation is that the presence of the industry in the neighbourhood can be perceived by residents as a threat to residents’ health. Families with asthmatic children may avoid living near a chemical plant or move away. Therefore, migration bias may have attenuated the observed associations with lung function and/or respiratory symptoms in our study population. On the other hand, concern about industry can increase the reported children’s respiratory symptoms when parents with a high risk perception are more likely to report the presence of respiratory symptoms in their children.

A fourth limitation is that we did not had complete information on all parent-children pairs. Selective response in the questionnaire may cause bias in reported associations. Sensitivity analyses were performed to evaluate possible change of the reported associations by comparing the analysis performed on persons with information about lung function with the analysis on cases with complete information on both lung function and respiratory symptoms. This sensitivity analysis revealed no meaningful influence of the restriction in study population on the associations between air pollution and lung function.

A fifth limitation is that the variability of different lung function measures is age dependent and a fixed cut-off of for low lung function (in this study defined as below 85% predicted) can therefore be inappropriate [25–27]. Therefore the internal reference equations were recalculated separately for children below and above the median age. The results showed similar outcomes.

## Conclusions

In this cross-sectional study modelled PM_2.5_ and NO_X_ exposure from an area with heavy industry was related to a significantly lower lung function in school children. The PM_2.5_ exposure was also significantly associated with presence of the respiratory symptom dry cough.

## Additional File

Additional file 1: **Figure S1.** Modelled NO_X_ isoconcentration contours (μg/m^3^), five years average exposure (2008–2012) without background concentration. Map reprinted from Kadaster [28] in the Netherlands under a CC-BY-4.0 license, 2017″. (JPEG 1976 kb)

## Abbreviations

FEV1: 1-s forced expiratory volume
FVC: Forced vital capacity
Low lung function: < 85% predicted.
MMEF: Maximum midexpiratory flow
NO_X_: Nitrogen oxides
OR: Odds ratio
PEF: Peak expiratory flow
PM: Particulate matter
PM_10_: Particulate matter with an aerodynamic diameter of 10 μm or less
PM_2.5_: Particulate matter with an aerodynamic diameter of 2.5 μm or less
SO_2_: Sulphur dioxide
