# Supplementary figures and images for: The effect of industry-related air pollution on lung function and respiratory symptoms in school children
Source: Environ Health. 2018 Mar 27;17:30. doi: 10.1186/s12940-018-0373-2 (PMC5872550; doi:10.1186/s12940-018-0373-2)

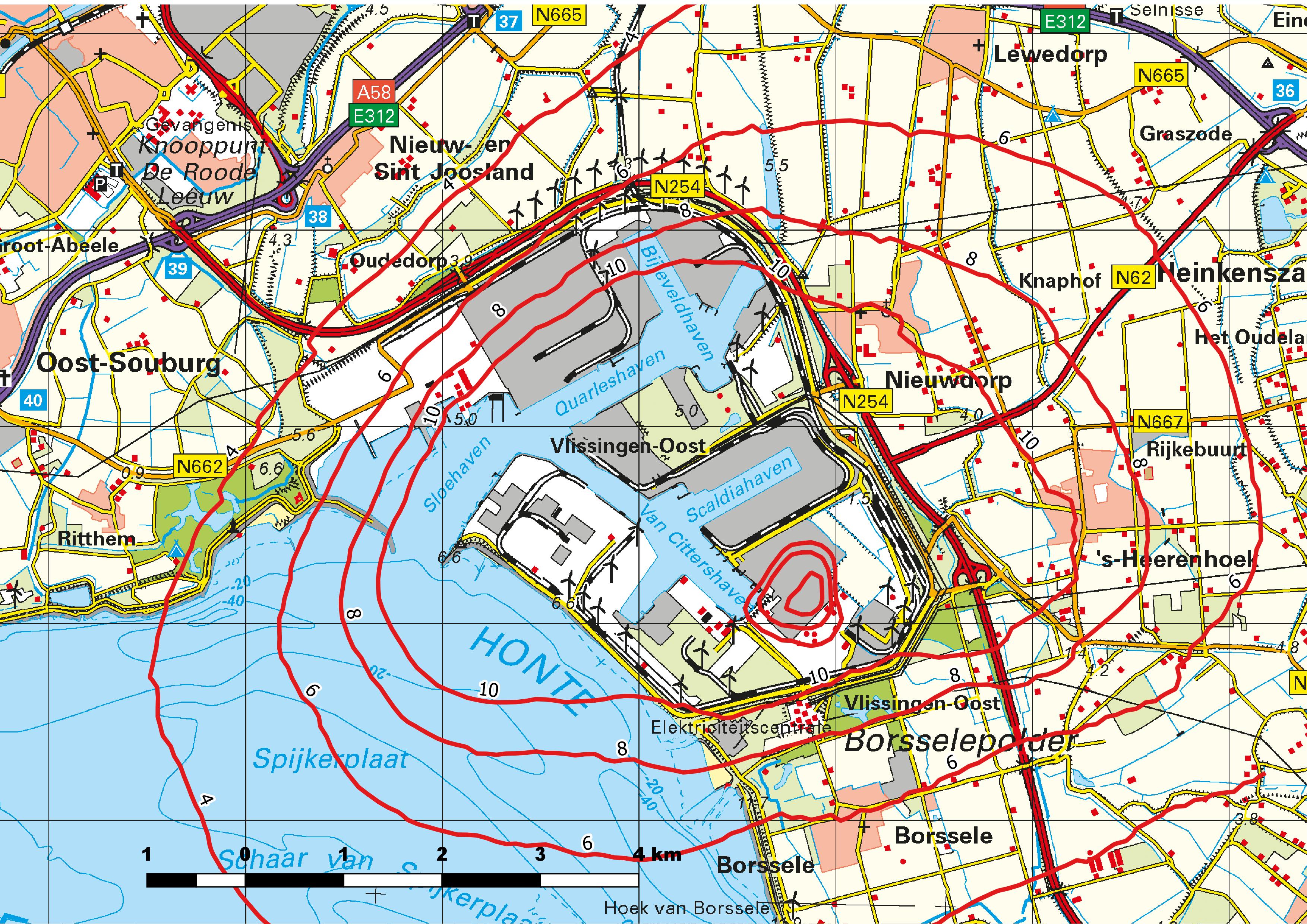

Supplement: Supplementary file 1 — Figure S1. Modelled NOX isoconcentration contours (μg/m3), five years average exposure (2008–2012) without background concentration. Map reprinted from Kadaster [28] in the Netherlands under a CC-BY-4.0 license, 2017″. (JPEG 1976 kb) [file 12940_2018_373_MOESM1_ESM.jpeg]
